# Center Variation in Intestinal Microbiota Prior to Late-Onset Sepsis in Preterm Infants

**DOI:** 10.1371/journal.pone.0130604

**Published:** 2015-06-25

**Authors:** Diana H. Taft, Namasivayam Ambalavanan, Kurt R. Schibler, Zhuoteng Yu, David S. Newburg, Hitesh Deshmukh, Doyle V. Ward, Ardythe L. Morrow

**Affiliations:** 1 Perinatal Institute, Cincinnati Children's Hospital, Cincinnati, Ohio, United States of America; 2 Department of Environmental Health, University of Cincinnati, Cincinnati, Ohio, United States of America; 3 Pediatrics, University of Alabama at Birmingham, Birmingham, Alabama, United States of America; 4 Biology, Boston College, Chestnut Hill, Massachusetts, United States of America; 5 Broad Institute, Cambridge, Massachusetts, United States of America; Columbia University, UNITED STATES

## Abstract

**Objective:**

Late onset sepsis (LOS) contributes to mortality and morbidity in preterm infants. We tested the hypotheses that microbes causing LOS originate from the gut, and that distortions in the gut microbial community increases subsequent risk of LOS.

**Study Design:**

We examined the gut microbial community in prospectively collected stool samples from preterm infants with LOS and an equal number of age-matched controls at two sites (Cincinnati, OH and Birmingham, AL), by sequencing the bacterial 16S rDNA. We confirmed our findings in a subset of infants by whole genome shotgun sequencing, and analyzed the data using R and LEfSe.

**Results:**

Infants with LOS in Cincinnati, as compared to controls, had less abundant *Actinobacteria* in the first samples after birth (median 18 days before sepsis onset), and less abundant *Pseudomonadales* in the last samples collected prior to LOS (median 8 days before sepsis onset). Infants with LOS in Birmingham, as compared to controls, had no differences identified in the first sample microbial communities, but *Lactobacillales* was less abundant in the last samples prior to LOS (median 4 days before sepsis onset). Sequencing identified detectable levels of the sepsis-causative organism in stool samples prior to disease onset for 82% of LOS cases.

**Conclusions:**

Translocation of gut microbes may account for the majority of LOS cases. Distortions in the fecal microbiota occur prior to LOS, but the form of distortion depends on timing and site. The microbial composition of fecal samples does not predict LOS onset in a generalizable fashion.

## Introduction

Late-onset neonatal sepsis (LOS) is a major cause of morbidity and mortality in preterm infants. LOS occurs in more than 20% of very low birth weight infants [1] and is associated with increased risk of mortality [2]. Survivors of LOS exhibit worse long-term outcomes, with increased risk of cerebral palsy, vision, and hearing impairment [3]. Given the seriousness of LOS, diagnostic and predictive biomarkers are needed [4].

Two-thirds of LOS cases in preterm neonates have been attributed to indwelling vascular catheters; this statistic is based on co-occurrence of events, i.e., catheter use within 48 hours of sepsis onset [5]. However, LOS is often caused by organisms that are typically found colonizing the intestinal tract, suggesting that LOS may often originate from translocation of gut organisms across the intestinal lining into the blood stream. Indeed, several small studies have demonstrated that the majority of LOS cases, in particular, those caused by group B *Streptococcus*, *Serratia marcescens*, or *Escherichia coli* strains, are due to organisms that were detectable in infant stool samples collected prior to the occurrence of sepsis [6, 7]. Several investigators have applied 16S microbial community analysis to examine distortions in the intestinal microbiota in advance of neonatal sepsis. A study of six infants found that those who later developed sepsis had an intestinal microbiota distinct from that of healthy infants [8]; samples from infants who later developed LOS tended to have more *Staphylococcus* and *Proteobacteria* than healthy infants who exhibited greater microbial diversity and more anaerobic bacteria [8]. Another study, of 10 LOS cases and 18 matched control infants, found a decrease in diversity and lower *Bifidobacteria* counts preceding LOS compared to control infants [9]. A third study, of 27 infants, found an increase in *Staphylococcaceae* associated with sepsis but did not report a difference in diversity between cases and controls [10].

The microbiota of the gut is a complex community, and dysbiosis within that community can result in disease. A clear example of this process is *Clostridium difficile* infection following antibiotic treatment: Antibiotic treatment may remove microbial community members that normally keep *C*. *difficile* in check, thereby allowing *C*. *difficile* to overgrow and cause disease. Restoration of the microbial community through fecal transplant is highly successful in treating refractory *C*. *difficile* disease [11]. Another example of microbial community dysbiosis predisposing to disease is seen in the different colonization patterns of preterm infants who develop necrotizing enterocolitis (NEC) compared to infants who remain healthy [12, 13]. The ability of a healthy microbial community to prevent disease may have implications for LOS. Indeed, mouse studies have demonstrated that microbiota are important to the barrier function of gastrointestinal epithelium and that alterations to microbiota influence intestinal permeability [14].

We therefore hypothesized that the composition of the intestinal microbiome influences the risk of LOS in premature infants. This study addresses two distinct but related questions: 1) What proportion of neonatal LOS are potentially attributable to translocation of gut microbes; and 2) is there an identifiable pattern of microbial community dysregulation that occurs in advance of neonatal sepsis?

## Methods

### Subjects

Study infants were selected from all infants <29 weeks gestational age who were enrolled in an ongoing cohort study of novel biomarkers for NEC. Infants were enrolled from three level III Neonatal Intensive Care Units (NICUs), including one in Birmingham, AL and two in Cincinnati, OH. LOS was defined as development of blood culture-proven sepsis more than72 hours after birth. When blood culture indicated the cause of sepsis as coagulase-negative *Staphylococcus* or diphtheroid *Bacilli*, infants had to have been either blood culture positive for an additional organism or treated with antibiotics for at least 5 days beginning within 72 hours of the time of the blood draw for the culture. To be eligible for inclusion as a case, infants had to have at least one stool sample collected and successfully processed prior to sepsis onset. Eligibility for selection as a control required that an infant was born during the time period between the birth of the first case and the birth of the last case at each site, and survive free of proven NEC and sepsis. Additionally, to be eligible as a control, an infant had to have at least one successfully sequenced stool sample. Controls were frequency matched to cases on study site and gestational age. The Cincinnati Children’s Hospital Medical Center IRB, the University of Alabama IRB, the TriHealth IRB, and the University of Cincinnati IRB approved this study. Written consent was obtained from parents or guardians of study subjects.

### Sample Collection

Serial stool samples were collected from infants on scheduled collection postnatal days 5, 8, 11, 14, and 21 plus or minus two days. For this study, the first successfully sequenced stool sample and the last successfully sequenced sample prior to LOS onset from each infant were included. For the last sample analysis, control samples were frequency matched to case samples on collection postnatal days. Frequency matching on the postnatal day of sample collection was done to account for the known normal progression of intestinal microbiota that occurs with increasing time since birth [15]. Samples were collected from soiled diapers, immediately refrigerated at 4°C in the NICU and transported to the laboratory where they remained in the refrigerator until processing and storage at -80°C. All Birmingham samples were stored without buffer, as facilities for processing samples were limited. In Cincinnati, most samples were processed and stored with thioglycollate buffer. However, the protocol for use of buffer changed over time as efforts were made to optimize sample storage: Thus, for the first sample analysis, 4 of 22 control samples and 7 of 22 case samples were stored without thioglycollate (Fisher’s Exact Test, p = 0.49). In Cincinnati for the last sample analysis, 1 of 22 control samples and 5 of 22 case samples were stored without thioglycollate (Fisher’s Exact Test, p = 0.19). From previous methodologic studies, we found that samples stored without thioglycollate to be enriched in phylum *Bacteroidetes*, *Proprionibacterineae* (a family belonging to class Actinomycetales), and *Leuconostocaeae* (a family belonging to class Bacilli)[16].

### Stool Extraction and 16S rDNA Sequencing

Stool extractions were completed using the methods described in Morrow et al. 2013 [12]. The DNA was then sequenced and processed as described in Taft et al. 2014 [16]. The OTU table was rarified to 2000 reads per sample for analysis.

### Whole Genome Sequencing (WGS)

For half of the sepsis cases, sequence data was available to determine whether the sepsis-causing organism identified by the clinical laboratory was also present in the infant’s intestinal tract, as determined by WGS. The WGS data consisted of 101-base, paired-end reads on the Illumina HiSeq2000 platform producing an average of 3.2E+7 reads per sample. A minimal read length filter of 80nt was applied to all data used in analysis. Relative taxonomic abundances were determined from WGS data using MetaPhlAn v2.0 (https://bitbucket.org/biobakery/metaphlan2) [17].

### Statistical Analysis

Analysis focused on the 16S sequencing data as the number of cases and controls with WGS data was limited. Ordination was conducted using non-metric multi-dimensional scaling (NMDS),which did not indicate distinct microbial communities between the two sites, but the linear discriminant analysis effect size tool, LEfSe [18], found specific differences by site, as reported previously for this cohort [16](S1 and S2 Figs) The two Cincinnati NICUs had similar colonization patterns, and so were combined into one analysis. The initial analyses used the first available stool sample from each infant prior to LOS. We then analyzed the last sample prior to sepsis in cases compared to a sample at a matched postnatal timing from controls. To select the appropriate sample from controls for this analysis, the sample collection day of life was frequency matched to the sample collection day of life for the sepsis cases by sampling without replacement. We first created a table including the day of life of collection of all samples from control infants and a table of collection day of life of the last sample prior to sepsis for all case samples. The first control sample was selected by identifying the collection day of life of a case sample on which the fewest control samples were available. Those two samples and all additional samples from that control and case infants were then removed from the pool eligible for selection. The selection of the next control sample was made by again identifying the collection day of life of a case sample with the fewest control samples to choose from. This procedure was repeated until only a single case sample and a single control infant remained. The control sample collected closest to the collection day of life of the final case sample was selected for inclusion.

Alpha diversity was calculated using the Chao1 index and the Simpson index with the Vegan package in R; the Kruskal-Wallis test was used to test for differences in alpha diversity between cases and controls. Beta diversity was examined using non-metric multidimensional scaling, based on weighted and unweighted Uni-Frac distance as described in Morrow et al. [12] The linear discriminant analysis effect size tool (LEfSe) was used to screen for differences at all taxonomic levels between the LOS case and control samples [18]. Classification trees were generated to provide additional information on differences in microbial composition between the two sites. To generate trees for the Cincinnati analysis, the rpart package in R [19] was used with default settings. For Birmingham, the minimum number to split changed from 20 to 10 due to the small number of subjects. Logistic regression using a forward inclusion approach conducted in R was used in combination with the taxa difference cut-points identified by the classification tree, to test for significance of the models created by the classification trees. Covariates considered for inclusion in the model were delivery mode, sex, gestational age, birthweight, birth length, history of chorioamnionitis, infant exposure to antibiotics in the first week of life, mother’s marital status, preeclampsia, and gravida. In Cincinnati, extraction protocol and thioglycollate storage protocol were also considered. Race was not included in the model because there were no differences in race between cases and controls in Birmingham, and in Cincinnati controls had only one more infant identified as black than did controls. Since the number of subjects included at each site was small, the models of Cincinnati data were limited to 4 covariates at a time and the models of Birmingham data were limited to two covariates at a time. All models are reported for sites and time points with multiple models possible.

Finally, we analyzed the most common cause of LOS, coagulase-negative *Staphylococcus* (CONS), compared to all controls to determine whether LOS caused by a single organism has a distinct form of dysbiosis. No other cause of LOS was numerous enough to analyze as a subgroup.

## Results

### Study subjects

Included for study were 13 infants in Birmingham, AL and 20 infants in Cincinnati, OH who developed LOS and had at least one successfully sequenced stool sample that was collected prior to disease onset. Also included were an equal number of control infants who did not develop LOS. Controls were frequency matched to cases by site and gestational age. LOS cases and controls were well matched (Table 1) and we noted no significant differences between cases and controls at either site in any clinical or demographic variables. In Birmingham, the mean birth weight was 736 grams for infant with LOS and 804 grams for control infants. In Cincinnati, the mean birth weight was 795 grams for infant with LOS and 851 grams for controls. The median gestational age at birth was 24 weeks for Birmingham cases, 25 weeks for Birmingham controls and Cincinnati cases, and 25.5 weeks for Cincinnati controls. However, potentially important differences were noted between sites: Intrapartum antibiotics were given to 92% of Birmingham mothers, whereas in Cincinnati, intrapartum antibiotics were given to 75% of mothers. Infant antibiotic use was significantly higher in Birmingham than Cincinnati (p = 0.051, Kruskal-Wallis test). Furthermore, the rate of infant formula use was lower in Cincinnati (where none received formula prior to day of life 15) compared to Birmingham (p = 0.005, Fisher’s exact test).

**Table 1:** Comparison of cases and controls in Birmingham and Cincinnati.

Characteristic	Birmingham		Cincinnati	
	Cases	Controls	Cases	Controls
	n = 13	n = 13	n = 20	n = 20
Multiple Birth	3 (23%)	2 (15%)	8 (40%)	5 (25%)
C-section	5 (38%)	6 (46%)	11 (55%)	11 (55%)
Sex, female	7 (54%)	7 (54%)	7 (35%)	11 (55%)
Hispanic	1 (8%)	0 (0%)	2 (10%)	0 (0%)
Race, Black, other	7 (54%), 0 (0%)	7 (54%), 0 (0%)	7(35%), 0(0%)	9(45%), 1(5%)
Maternal antibiotics given	12 (92%)	12 (92%)	17 (85%)	13 (65%)
Infant birthweight (mean), g	736	804	795	851
Infant gestation, weeks (median)	24	25	25	25.5
Days on antibiotics in first 14 (median)*	6	8	6	7
Any formula prior to last included stool sample**	5 (38%)	4 (31%)	2 (10%)	0 (0%)

Comparing all subjects between sites:

*p = 0.05;

**p = 0.005

Twelve (92%) of 13 infants with LOS in Birmingham had Gram-positive sepsis (median day of life 13 for sepsis onset) and 1 had Gram-negative sepsis on day of life 12. Twelve (60%) of 20 infants with LOS in Cincinnati had Gram-positive sepsis (median day of life 16) and 6 (30%) had Gram-negative sepsis (median day of life 33), while 2 (10%) infants were culture positive for both Gram-positive and Gram-negative organisms at the time sepsis was diagnosed (Table 2). To examine the concordance between the clinical LOS isolate and the presence of a matching organism in infant stool, we utilized whole genome sequencing data that was available for samples prior to sepsis in 17 of the 33 cases. If a species-level match was identified from the stool metagenome, we considered it a match to the clinical isolate. For example, if the causative organism was identified as coagulase negative *Staphylococcus*, we considered a match to occur if the stool metagenome indicated the presence of at least one species of coagulase negative *Staphylococcus*. If no information below the genus level was available from the clinical laboratory, we considered a match to have occurred if DNA from at least one species belonging to that genus was detected in stool. Of these 17 cases, 14 (82%) had at least 1 stool sample with identifiable abundance of the causative organism as identified by the clinical laboratory; results matched to the lowest taxonomic level for which clinical and stool data were available (genus or species, Table 2). The three infants whose clinical isolate was not matched by identifying the same organism in stool sample had LOS caused by *Klebsiella pneumoniae*, Group B *Streptococcus*, or *Staphylococcus aureus*.

**Table 2:** Sepsis cause, day of life of blood collection, and the number of stool samples collected prior to sepsis.

Case No.	City	Causative Organisms identified by clinical laboratories	Day of life of blood culture	No. samples prior to sepsis	Closest related organism identified in WGS data	Scored as match to cultured isolate^
1	C	Coagulase negative *Staphylococcus*	13	1	*Staphylococcus epidermidis*	**Yes**
8	C	Coagulase negative *Staphylococcus*	10	3	*Staphylococcus epidermidis*	**Yes**
9	C	Coagulase negative *Staphylococcus*	20	3	*Staphylococcus epidermidis*	**Yes**
11	C	Coagulase negative *Staphylococcus*	13	2	*Staphylococcus epidermidis Staphylococcus hominis*	**Yes**
12	C	Coagulase negative *Staphylococcus*	29	2	*Staphylococcus epidermidis Staphylococcus warneri*	**Yes**
16	C	Coagulase negative *Staphylococcus*	13	2	*Staphylococcus epidermidis*	**Yes**
22	B	Coagulase negative *Staphylococcus*	9	1	*Staphylococcus epidermidis*	**Yes**
33	B	*Enterococcus faecalis*	21	2	*Enterococcus faecalis*	**Yes**
21	B	*Streptococcus* viridans	19	2	*Streptococcus thermophilus*	**Yes** *
5	C	Diphtheroid bacilli	19	1	*Corynebacterium striatum Corynebacterium pyruviciproducens*	**Yes**
2	C	*Enterobacter cloacae*	27	3	*Enterobacter cloacae*	**Yes**
13	C	*Escherichia coli*	20	5	*Escherichia coli*	**Yes**
15	C	*Escherichia coli*	35	3	*Escherichia coli*	**Yes**
4	C	*Klebsiella*	41	4	*Klebsiella* unclassified	**Yes**
14	C	*Staphylococcus aureus*	6	1	*Staphylococcus epidermidis*	No
7	C	Group B *Streptococcus*	27	1	*Streptococcus salivarius*	No
3	C	*Klebsiella pneumoniae*	31	4	*Klebsiella oxytoca Klebsiella* unclassified	No
No WGS data available prior to sepsis						
6	C	*Serratia marcescens Enterococcus faecium Enterococcus faecalis*	64	1		
10	C	Group B *Streptococcus*	28	2		
17	C	Group B *Streptococcus*	72	1		
18	C	*Escherichia coli*	38	1		
19	C	*Pseudomonas* Coagulase negative *Staphylococcus*Group B *Streptococcus*	27	2		
20	C	Coagulase negative *Staphylococcus*	13	1		
23	B	*Enterococcus faecalis*	18	2		
24	B	Coagulase negative *Staphylococcus*	9	1		
25	B	*Escherichia coli*	12	2		
26	B	Coagulase negative *Staphylococcus*	10	1		
27	B	*Enterococcus faecalis*	14	2		
28	B	*Staphylococcus aureus* (methicillin resistant)	9	1		
29	B	Coagulase negative *Staphylococcus*	40	2		
30	B	*Staphylococcus aureus* (methicillin resistant)	105	2		
31	B	Coagulase negative *Staphylococcus*	11	1		
32	B	Coagulase negative *Staphylococcus*	12	2		

*Streptococcus viridans is a group of organisms that includes Streptococcus thermophilus.

^^^WGS results were scored as a match to the clinical isolate if the same taxonomy was identified at the species level, or if the species identified by WGS was an member of the genus noted clinically when clinical results were not conclusive at the species level.

### Microbial Diversity

To examine the microbial community pattern prior to LOS, we examined alpha and beta diversity in the first sample after birth and the last sample available prior to LOS onset.

#### First Sample Analysis

The timing of first sample did not differ significantly between cases and controls within or between Birmingham and Cincinnati sites. For Birmingham infants, the median sample collection day of life was day 7 (range, 4–10) for LOS cases and day 8 (range, 6–12) for controls; the median number of days from first sample collection day until sepsis onset was 8 days (range, 1–96). For Cincinnati infants, the median sample collection day of life was day 8 (range, 3–20) for LOS cases and day 8 (range, 4–21) for controls; the median number of days from first sample collection day until sepsis onset was 18 days (range was 2–56).

In Birmingham, cases had a significantly (p<0.02) lower alpha diversity than controls by both the Simpson and Chao1 metrics. In Cincinnati, however, there were no significant differences by either alpha diversity index between cases and controls. For both Birmingham and Cincinnati analyses, there was no clustering of cases or controls in the non-metric multidimensional scaling ordinations, whether using weighted or unweighted UniFrac measures (data not shown.)

**Fig 1:**
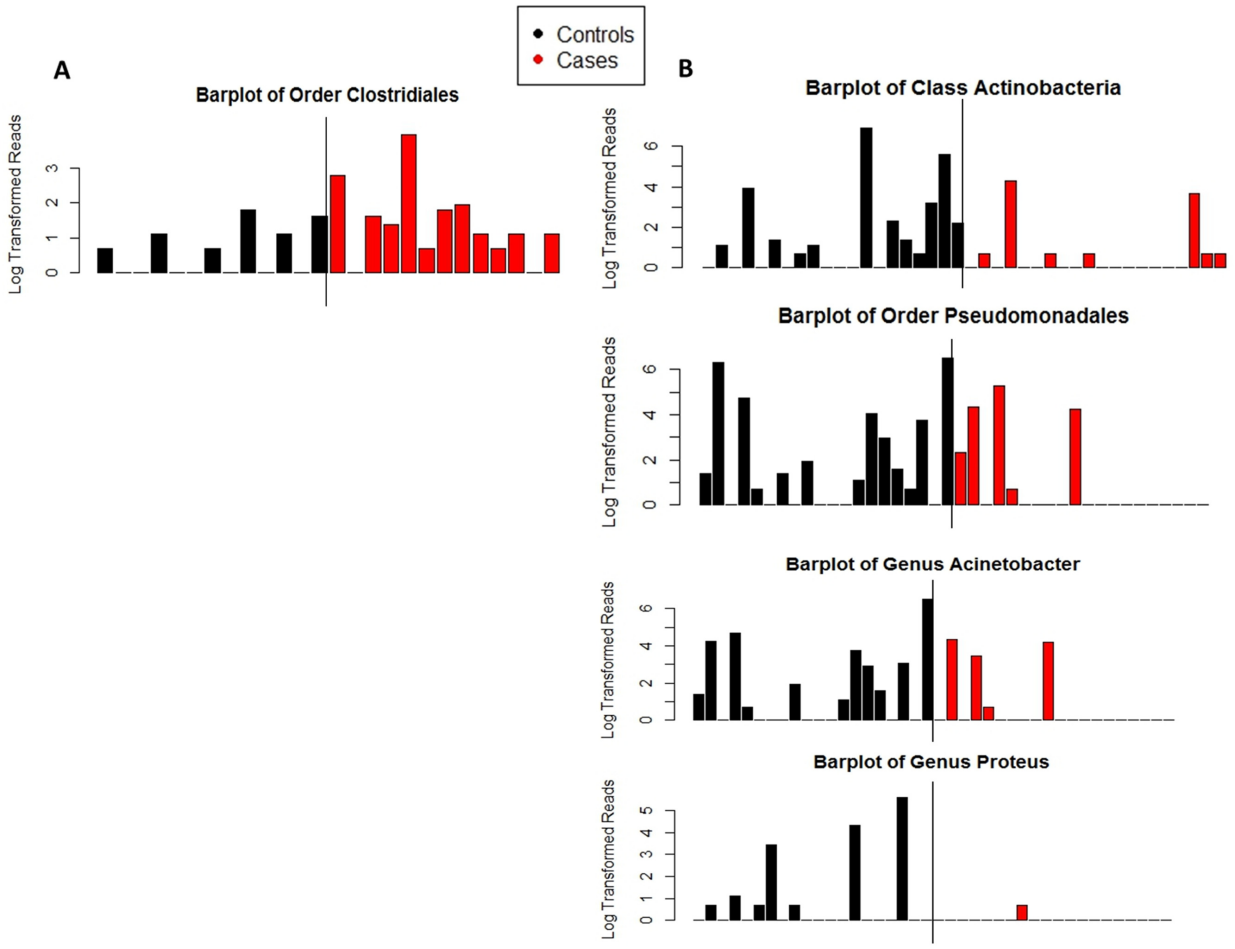
Barplots of taxa identified as differential abundant in cases and controls. Y-axis was transformed by taking the base ten logarithm of the number of reads plus one. Samples were rarefied to 2000 reads per sample prior to creating the plots. A) Taxa differentially abundant by LEfSe in the first sample analysis in Birmingham. Median number of reads of *Clostridiales* in controls was 0, range was 0 to 5. Median number of reads of *Clostridiales* in cases was 2, the range was 0 to 50. Class *Clostridia* was also significantly different between cases and controls, it is not shown here because class *Clostridia* and order *Clostridiales* had a correlation of 1. B) Taxa differentially abundant by LEfSe in the first sample analysis in Cincinnati. Median number of reads of class *Actinobacteria* in controls was 1.5, range was 0 to 985. Median number of reads of class *Actinobacteria* in cases was 0, range was 0 to 74. Median number of reads of *Pseudomonadales* in controls was 2.5, range was 0 to 661. Median number of reads of *Pseudomonadales* in cases was 0, range was 0 to 193. Median number of reads of *Acinetobacter* in controls was 1.5, range was 0 to 660. Median number of reads of *Acinetobacter* in cases was 0, range was 0 to 75. Median number of reads of *Proteus* in controls was 0, range was 0 to 267. Median number of reads of *Proteus* in cases was 0, range was 0 to 1, Phyla *Actinobacteria* was also significantly different between cases and controls, it is not shown here because it has a correlation 0.9996 with class *Actinobacteria* and was visually indistinguishable. Family *Moraxellaceae* was also significantly different between cases and controls; it is not shown here because it had a correlation of 1 with genus *Acinetobacter*.

**Fig 2:**
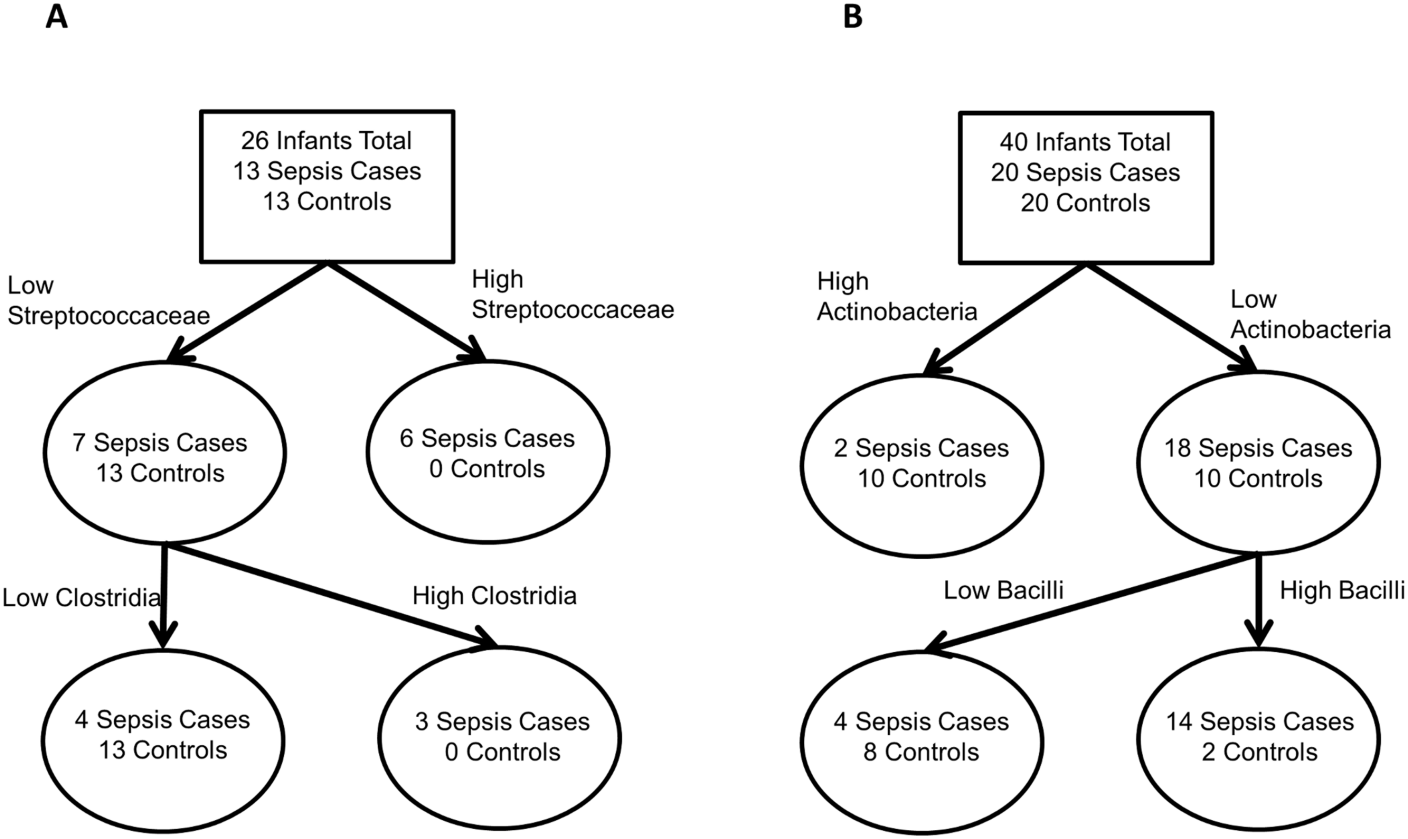
First sample classification tree results. (A) Birmingham results, showing infants colonized with *Streptococcaceae* and infants with higher levels of *Clostridia* are at greater risk of sepsis. (B) Cincinnati results, showing that infants colonized with greater relative abundance of *Actinobacteria* and infants with lower relative abundance of *Bacilli* are protected from sepsis.

For Birmingham samples, LEfSe analysis indicated a significant association of *Clostridia* and *Clostridiales* abundance in infants who later developed LOS compared to control infants (Fig 1A). *Clostridia* and *Clostridiales* more frequently colonized cases and were present in higher abundance. The classification tree model confirmed that *Clostridia* differed between cases and controls (cases were more likely to have greater than 0.3% relative abundance of *Clostridia*), but also found a signal from *Streptococcaceae* (cases were more likely to have greater than 0.1% relative abundance of Streptococcaceae (Fig 2A). The misclassification rate of the tree was 15%. However, logistic regression models did not find a significant association between the cut points identified in the classification tree and sepsis. The final model did not include any other covariates, all dropped out of the model as non-significant (model not shown).

For Cincinnati samples, LEfSe analysis indicated a statistically significant association between higher levels of phylum *Actinobacteria* and genus *Proteus* in control infants compared to LOS infants. In addition, control infants were more likely to be colonized with order *Pseudomonadales*, family *Moraxellaceae*, and genus *Acinetobacter* (Fig 1B). As with the Birmingham samples, examination of the differential feature plot (not shown) found the differences observed were not driven by outliers. The classification tree confirmed that phylum *Actinobacteria* differed between cases and controls, and detected a further signal. In the subset of infants lacking *Actinobacteria*, the classification tree found that infants whose stool samples contained at least 3% *Bacilli* by relative abundance (65 of 2000 rarefied reads) were more likely to be cases than were infants whose stool samples had fewer reads (Fig 2B). The misclassification rate of the tree in Cincinnati was 20%. Logistic regression was again used to determine the significance of the cut points identified in the classification tree at p<0.05; only the *Actinobacteria* cut point was significant. All covariates dropped out of the model as non-significant (Table 3).

**Table 3:** Logistic modeling results of LOS by site using cut points defined by classification trees.

Model	Other covariates+	Independent Variables	beta	p-value
Cincinnati—first sample	none	***Actinobacteria* > = 0.1%**	**-2.67**	**0.018**
		*Bacilli* < 3.25%	-1.39	0.39
Cincinnati—last sample	Gravida (p = 0.098)	***Pseudomonadales* > = 0.1%**	**-2.65**	**0.029**
		***Clostridiaceae* > = 0.35%**	**2.43**	**0.030**
		***Proteobacteria* < 72.3%**	**-2.59**	**0.011**
Birmingham—last sample Model 1	Infant GA (p = 0.087)	***Lactobacillales* present**	**-3.6**	**0.017**
Birmingham—last sample Model 2	Chorioamnionitis (p = 0.070)	***Lactobacillales* present**	**-3.2**	**0.010**

Modeling confirmed the statistical significance of cut points identified from classification tree analysis except for the Birmingham first sample tree (not shown) and the unknown *Bacillales* in the Birmingham last sample tree.

^+^Covariates that remained in each model are noted along with their p-value.

#### Last Sample Analysis

There were no significant differences in day of life of sample collection in either Birmingham or Cincinnati. In both Birmingham and Cincinnati samples, one-third of samples included in the last sample analysis were also included in the first sample analysis. In Birmingham, the median sample collection day of life was 10 days (range, 4–14) for both LOS cases and controls; the median number of days from last sample collection day of life until sepsis onset was 4 days (range, 1–91). In Cincinnati, the median sample collection day of life was 13 days (range, 4–21) for both cases and controls; the median number of days from last sample collection day of life until sepsis onset was 8 days (range, 1–56). In Birmingham, cases had a significantly (p<0.001) lower alpha diversity than controls by the Simpson index but no significant difference by the Chao1 index. As in the first sample analysis, there were no significant differences between cases and controls in alpha diversity by either metric in Cincinnati. There was no visible separation between cases and controls on the non-metric multidimensional scaling ordinations using either UniFrac metric at either site (data not shown.)

**Fig 3:**
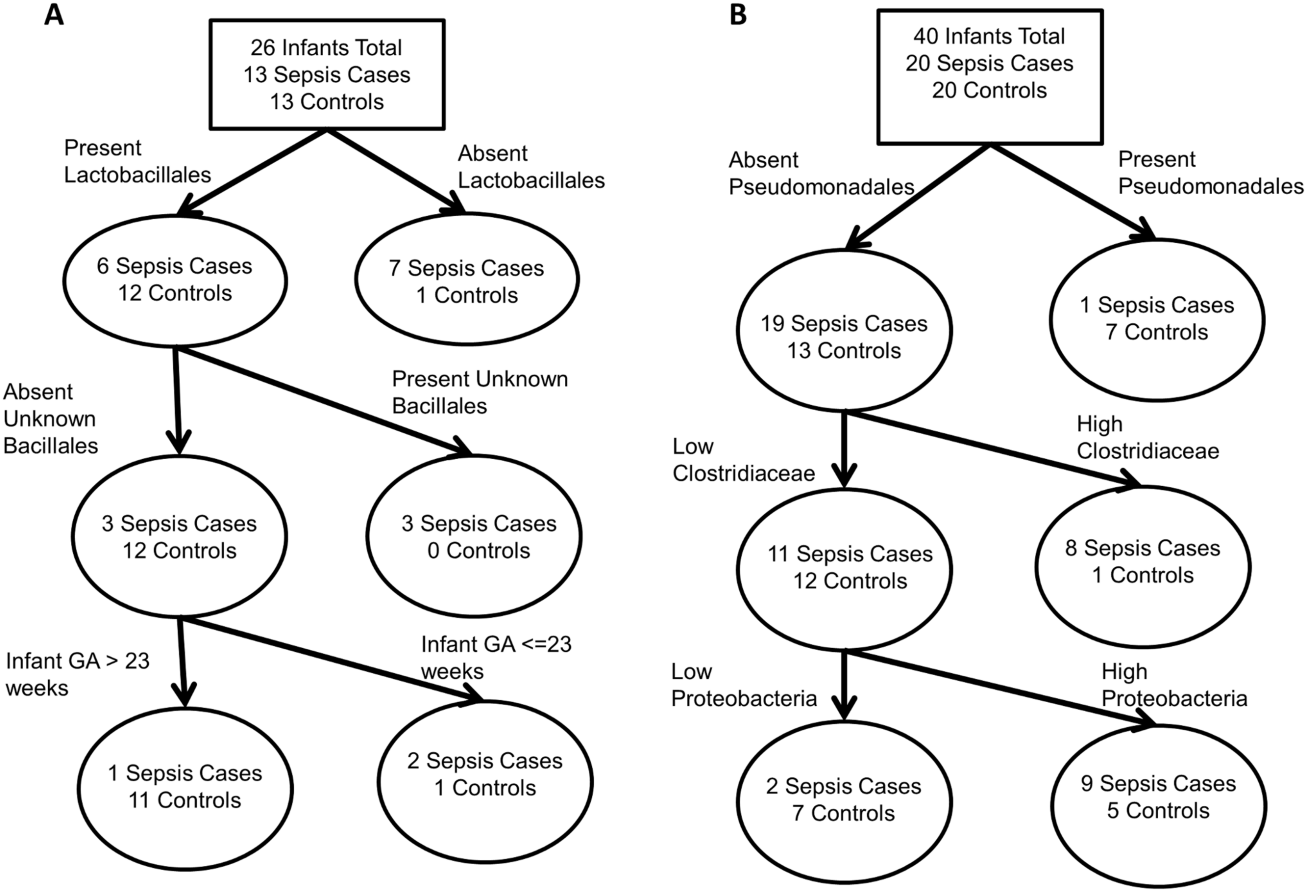
Last sample classification tree results for (A) Birmingham showing infants without *Lactobacillales*, with the unknown OTU of *Bacillales*, or born at younger gestational ages were more at risk of sepsis and (B) Cincinnati showing that infants with high levels of *Enterobacteriales* or high levels of *Firmicutes* were at increased risk of sepsis.

For Birmingham, LEfSe did not detect significant differences in microbial colonization above the OTU level between cases and controls. The OTUs enriched in both cases and controls were almost entirely classified as *Enterobacteriaceae* (data not shown). A classification tree found that the presence or absence of *Lactobacillales*, the presence or absence of an OTU of *Bacillales* of unknown family, and infant gestational age less than or equal to 23 weeks were the best predictors of sepsis status (Fig 3A). The misclassification rate was 12%. Logistic regression confirmed the significance of the presence of *Lactobacillales* as protective against sepsis, but the unknown Bacillales had a p-value >0.99 in all possible models and so was excluded. Infant gestational age (model 1) and chorioamnionitis (model 2) were borderline significant (Table 3).

**Fig 4:**
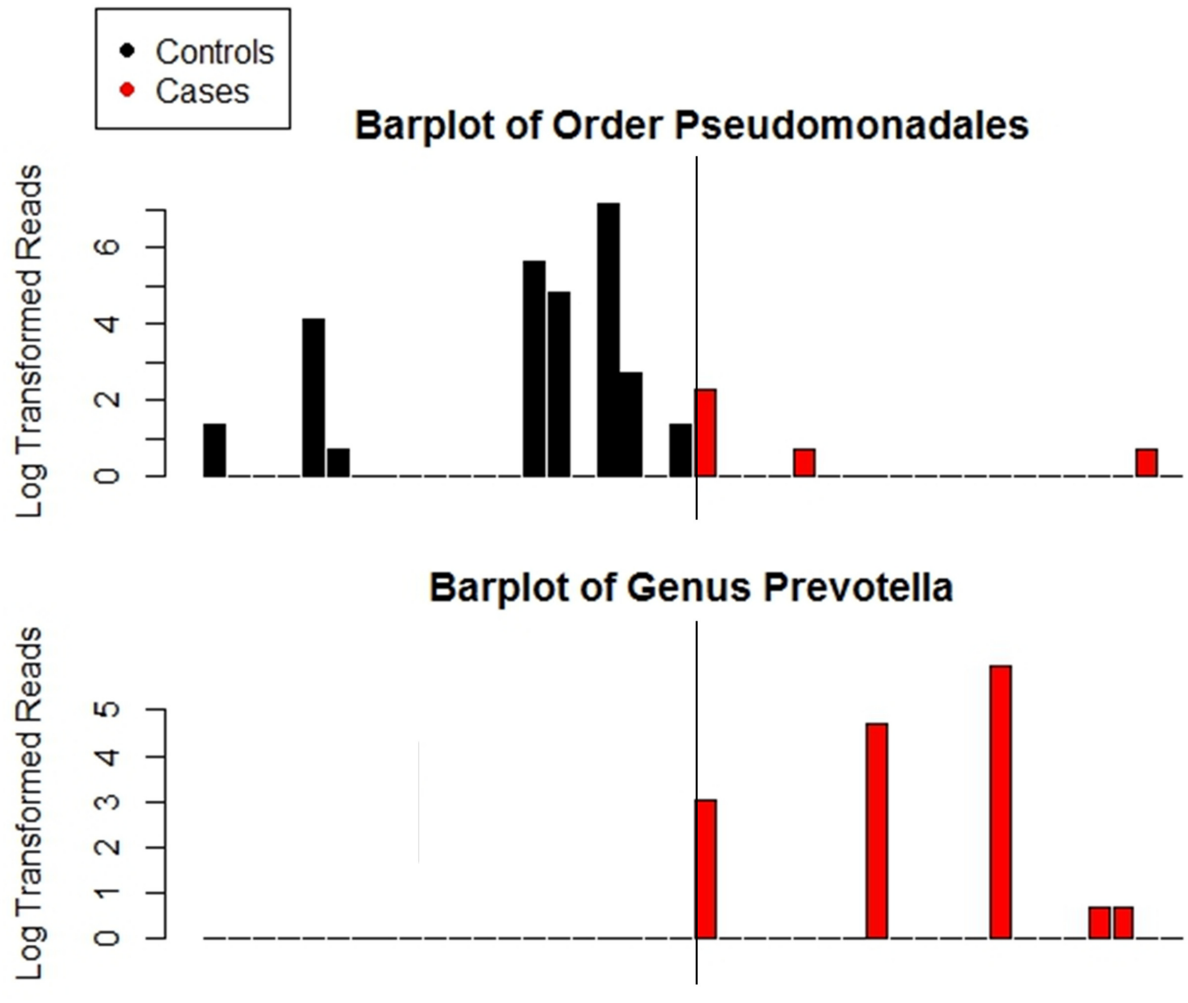
Barplots of taxa identified as differential abundant in cases and controls at genus level and higher in Cincinnati. Y-axis was transformed by taking the base ten logarithm of the number of reads plus one. Samples were rarefied to 2000 reads per sample prior to creating the plots. Median number of reads of *Pseudomonadales* in controls was 0, range was 0 to 1251. Median number of reads of *Pseudomonadales* in cases was 0, range was 0 to 9. No control samples had reads of *Prevotella*. Median number of reads of *Prevotella* in cases was 0, range was 0 to 384. Family *Prevotellaceae* was also significantly different between cases and controls, it is not shown here because it had a correlation of 1 with genus *Prevotella*. All other differences identified by LEfSe were at the OTU level. All differences identified by LEfSe in the Birmingham last sample analysis were at the OTU level.

For Cincinnati, we observed a significant association of *Prevotellaceae*, and *Prevotella* with cases and *Pseudomonadales* with controls (Fig 4). Examination of the differential feature plots revealed that *Prevotella* and *Prevotellaceae* were detected in 5 (25%) of the cases and never in controls. *Pseudomonadales* was present in in only 3 (15%) cases and 8 (40%) controls. A classification tree agreed with the *Pseudomonadales* finding, splitting first on *Pseudomonadales* but then split on *Clostridiaceae* and *Proteobacteria* instead of either *Prevotellaceae* or *Prevotella* (Fig 3B). The misclassification rate was 23%. Logistic regression confirmed that the cut points created by the classification tree were significant predictors of sepsis after controlling for the borderline significant covariate, gravida (Table 3).

### Cause-specific analysis of LOS dysbiosis

To examine the possibility that dysbiosis prior to sepsis is influenced by the causative organism, we focused on coagulase negative *Staphylococcus* (CONS), which constituted 42% of all LOS cases in our study, and was the most abundant causative organism. In the first sample after birth, the Birmingham CONS cases exhibited higher levels of *Actinobacteria* than in controls; but no other differences were identified between the groups. Whereas, analysis of first samples of Cincinnati CONS cases found lower *Actinobacteria* in cases than controls (S3 Fig) Analysis of last samples of Cincinnati CONS cases versus controls demonstrated enrichment in *Prevotella* and *Prevotellaceae*. Overall, the stool microbiome patterns identified with CONS cases are similar to those found for LOS cases overall. Staphylococcaceae were not found more abundantly in infant stool samples prior to CONS-associated sepsis.

### Analysis using whole genome sequencing

Though we had whole genome sequencing available only for a subset of Cincinnati LOS cases and controls, we examined the WGS data to determine consistency with 16S results. For the first sample analysis, we obtained WGS data for 12 LOS and 15 controls. Controls were enriched in *Actinomycetales* (an order of phylum *Actinobacteria*), *Streptococcus*, and *Streptococcaceae*. *Actinomycetales* enrichment is consistent with the 16S results showing enrichment of phylum *Actinobacteria*. WGS evidence of *Streptococcus* enrichment was not identified in the 16S data; it is, however, consistent with the 16S classification tree results insofar as *Streptococcus* belongs to class *Bacilli*. For the last sample analysis, WGS data were available for 10 LOS and 11 control samples. Similar to the 16S analysis, we found the *Prevotella* and *Prevotellaceae* enriched in cases compared to controls. Altogether, while 16S and WGS results were not identical, they were generally concordant.

## Discussion

Late onset sepsis in preterm infants is associated with very high mortality [20] and disproportionately high morbidities among survivors [21–23], including poor neurodevelopmental outcomes [3]. While the source of microbes causing LOS is not well established, reports suggest that the gut is often the source of microbes causing LOS in preterm neonates [7]. We used high-throughput sequencing to address the relationship between gut colonization and neonatal LOS. Consistent with previous reports, we identified a concordance of 82% between the causative LOS organism and its identification in stool samples prior to LOS; previous studies have reported concordance ranging from 64% [7] to 95% [6]. In this and an earlier publication [16], we have reported differences in colonization between our two study sites [16], which was the basis for analyzing the gut microbiota at each site separately. Site-specific analyses identified differences in microbial-community patterns in advance of LOS compared to controls. In Cincinnati, we found lower levels of Actinobacteria in early samples from infants who later developed LOS compared to controls, similar to a report from Mai et al, in which lower levels of *Bifidobacteria*, a genus of the phylum *Actinobacteria*, were observed in infants who later developed LOS [9]. In Birmingham, we found lower microbial-diversity in infants with LOS compared to controls, similar to reports by Madan et al. [8] and Mai et al. [9]. In Birmingham, the presence of *Lactobacillales* in the last sample was associated with protection against LOS, whereas in Cincinnati, the presence of *Lactobacillales* in the first sample, but not the last sample, was associated with protection against LOS. Thus, while we found significant site-specific differences in gut-microbial patterns in advance of LOS, we failed to detect a specific and generalizable gut-microbial colonization pattern in advance of LOS. Furthermore, the observed differences were dependent not only on site, but also on the timing of sample collection. Previous studies at single sites have reported associations with gut microbiota prior to the onset of sepsis, but also like the current study, the reported associations are not consistent between sites: One study found an association between increased abundance of *Staphylococcaceae [10]* and sepsis, while another found an association between decreased abundance of *Clostridia* and sepsis [8]; and some, but not all studies reported lower alpha diversity in sepsis cases [8, 9].

What could explain the site-specific differences in the gut-microbial pattern in advance of LOS? We sought to minimize the potential for chance associations by reporting only significant differences found at the bacterial family level in most cases and genus level in cases where OTUs were successfully classified at the genus level. Acquisition of intestinal microbiota, which begins immediately after birth [24], is impacted by gestational age [25], delivery mode [26], the use of antibiotics [27], feeding mode (breast milk versus formula) [28], the neonate’s genetic background [29], and the circulating microbiota of the environment [16]. In this study, we used site-, age-, birth weight- and sex-matched-controls thus minimizing the impact of confounding variables. However, the sites did significantly differ in their use of infant formula and the use of antibiotics (both were higher in Birmingham). The failure to find a consistent form of intestinal dysbiosis in prior to LOS between the sites could thus be due to site differences in initiating and advancing feeds, use of breast milk and use of antibiotics. Differences in the environmental microbiota could also potentially explain site-specific forms of dysbiosis.

Preterm infants undergo frequent and abrupt shifts in microbial community composition [15]. We analyzed two time points—the first collected sample after delivery and last sample prior to LOS onset—which identified different associations with LOS, similar to findings reported by Mai et al. [9]. We therefore speculate that to observe consistent within and between-site differences between cases and controls, more frequent (e.g., daily) sampling may be needed.

The sepsis-causing organism is frequently detectable in stool prior to disease onset but such pathogens are not typically found in infants who remain healthy [7]. We speculate that these pathogens are able to colonize the intestinal tract due to dysbiosis of the microbiome, and that these distortions vary by pathogen. Therefore, we compared stool from control infants to that of infants with LOS due to coagulase negative staphylococcus, the most common cause of neonatal LOS in our study. These sepsis-associated microbes did not occur more abundantly in infant stool samples in advance of LOS compared to controls. We posit that microbial-composition of the stool samples may not accurately represent the adherent gut microbial composition in advance of sepsis. Indeed, several groups have demonstrated that stool microbiota correlates poorly with the adherent microbiota identified by intestinal biopsy of specific intestinal sites [30, 31]. As the microbes that translocate to cause disease are likely to be adherent [32], analysis of stool samples may not adequately reflect the relevant adherent microbes and their activity.

LOS has a diverse etiology, with coagulase negative Staphylococcus and Gram-negative microbes such as *E*. *coli* accounting for majority of the cases. This complexity in etiology presents a substantial challenge to our understanding of LOS and underscores the inherent heterogeneity in how the LOS is defined in preterm infants. It is suggested that LOS due to coagulase negative Staphylococcus originates from the skin/indwelling catheters [33], while Gram negative LOS is thought to reflect the microbial translocation across the gut epithelium. However, our data suggest that LOS due to coagulase negative Staphylococcus may in some cases originate in the gut. Nevertheless, LOS due to Gram negative bacteria reflects a distinct group, with different etiology and pathobiology [34], as compared to LOS due to coagulase negative Staphylococcus.

In conclusion, our findings support the hypothesis that neonatal LOS is frequently caused by organisms that translocate from the intestinal tract, as 82% of LOS causative organisms were identified in infant stool samples prior to LOS onset. Within each center, we found differences in infant microbial communities preceding LOS compared to control infants. However, the differences in microbiota between cases and controls were not consistent between the sites. Our data suggest that factors besides intestinal microbial composition may underlie the risk of LOS. As yet unexplored in the context of LOS is dysfunction or dysregulation of the microbiota, or most likely, of host-microbial interactions. Such functional studies are key to advancing the understanding of the microbiota prior to sepsis.

## Supporting Information

S1 Fig Non-metric dimensional scaling ordinations. Red dots indicate samples from Cincinnati infants and black dots indicate samples from Birmingham infants. Panel A) All f (both cases and controls) from the first sample analysis. Panel B) First samples from *controls only*. Panel C) All subjects (cases and controls) from the last sample analysis. Panel D) Last samples from *controls only*. There is no clear separation of samples from the two sites in either the first or the last sample analysis.
(TIF)

S2 Fig Differences identified by LEfSe between Cincinnati (green) and Birmingham (red) samples. Panel A) All first samples (cases and controls) were included in the analysis. Cincinnati infants had higher levels of *Lactobacillales* and *Enterococcaceae* among others. Panel B) All last samples (cases and controls) were included in the analysis. Birmingham infants had higher levels of *Bacteroidetes* among others.
(TIF)

S3 Fig Taxa identified as different in cases and controls by LEfSe in the coagulase negative *Staphylococcus* to all controls first sample comparison. A) Taxa identified as different between Birmingham coagulase negative *Staphylococcus* cases and controls. Phyla *Actinobacteria* was also significantly different between cases and controls, it is not shown here because it has a correlation 0.9996 with class *Actinobacteria* and was visually indistinguishable. Family *Corynebacteriaceae* was also significantly different, it is not shown here because it has a correlation of 1 with genus *Corynebacterium*. Family *Moraxellaceae* was also significantly different between cases and controls, it is not shown here because it had a correlation of 1 with genus *Acinetobacter*. B) Taxa identified as different between Cincinnati coagulase negative *Staphylococcus* cases and controls. Phyla *Actinobacteria* was also significantly different between cases and controls, it is not shown here because it has a correlation 0.9996 with class *Actinobacteria* and was visually indistinguishable. Family *Corynebacteriaceae* was also significantly different, it is not shown here because it has a correlation of 1 with genus *Corynebacterium*. Family *Moraxellaceae* was also significantly different between cases and controls, it is not shown here because it had a correlation of 1 with genus *Acinetobacter*.
(TIF)

S1 File Metadata associated with all samples included in this study. (CSV)

S2 File Metadata dictionary. File contains variable name and description of variable for all variables included in the metadata file.
(CSV)

S3 File Rarefied OTU table containing OTU counts for all samples included in this study. (XLSX)
