# Supplementary figures and images for: Center Variation in Intestinal Microbiota Prior to Late-Onset Sepsis in Preterm Infants
Source: PLoS One. 2015 Jun 25;10(6):e0130604. doi: 10.1371/journal.pone.0130604 (PMC4482142; doi:10.1371/journal.pone.0130604)

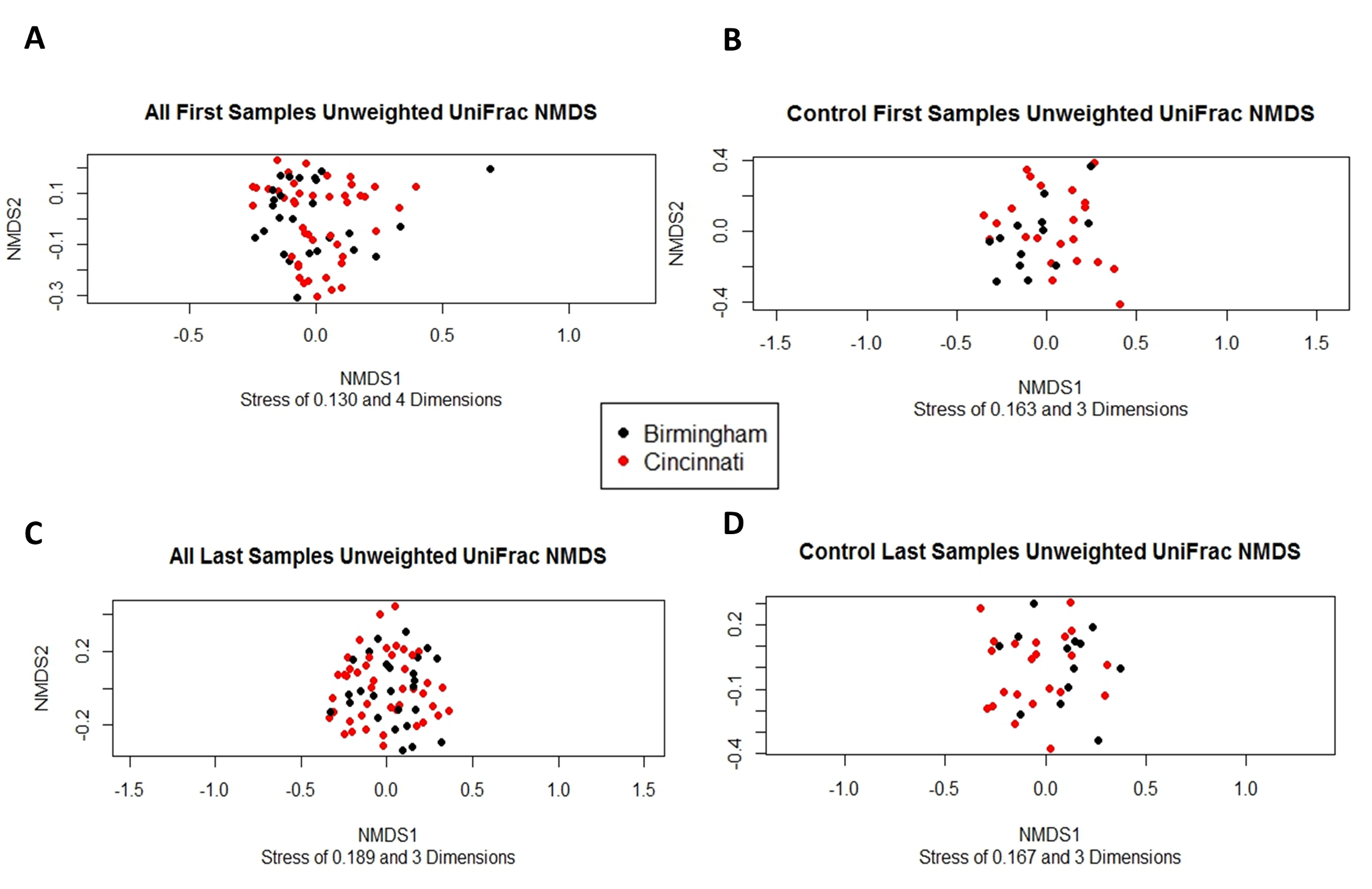

Supplement: S1 Fig — Red dots indicate samples from Cincinnati infants and black dots indicate samples from Birmingham infants. Panel A) All f (both cases and controls) from the first sample analysis. Panel B) First samples from controls only. Panel C) All subjects (cases and controls) from the last sample analysis. Panel D) Last samples from controls only. There is no clear separation of samples from the two sites in either the first or the last sample analysis. (TIF) [file pone.0130604.s001.tif]

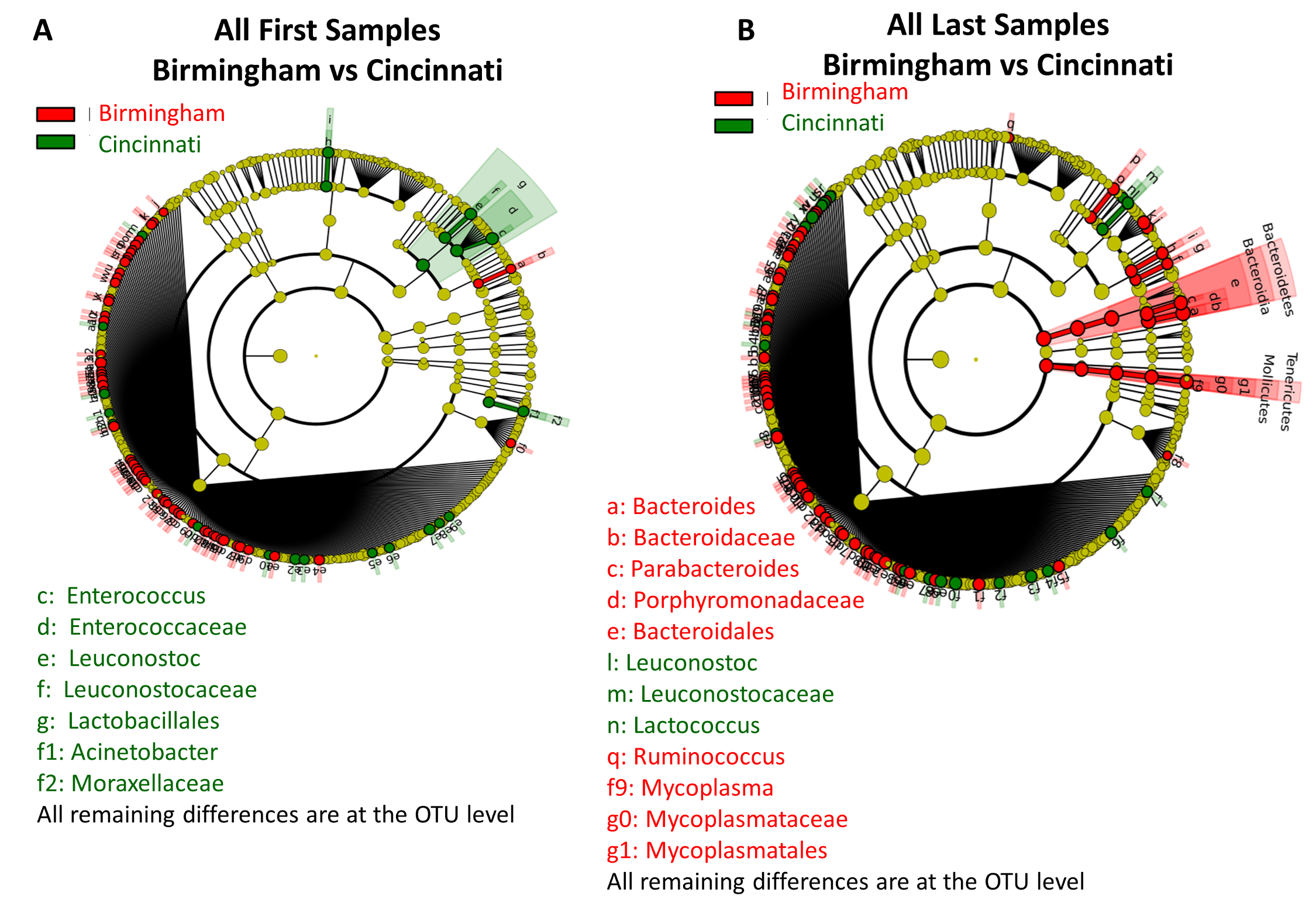

Supplement: S2 Fig — Panel A) All first samples (cases and controls) were included in the analysis. Cincinnati infants had higher levels of Lactobacillales and Enterococcaceae among others. Panel B) All last samples (cases and controls) were included in the analysis. Birmingham infants had higher levels of Bacteroidetes among others. (TIF) [file pone.0130604.s002.tif]

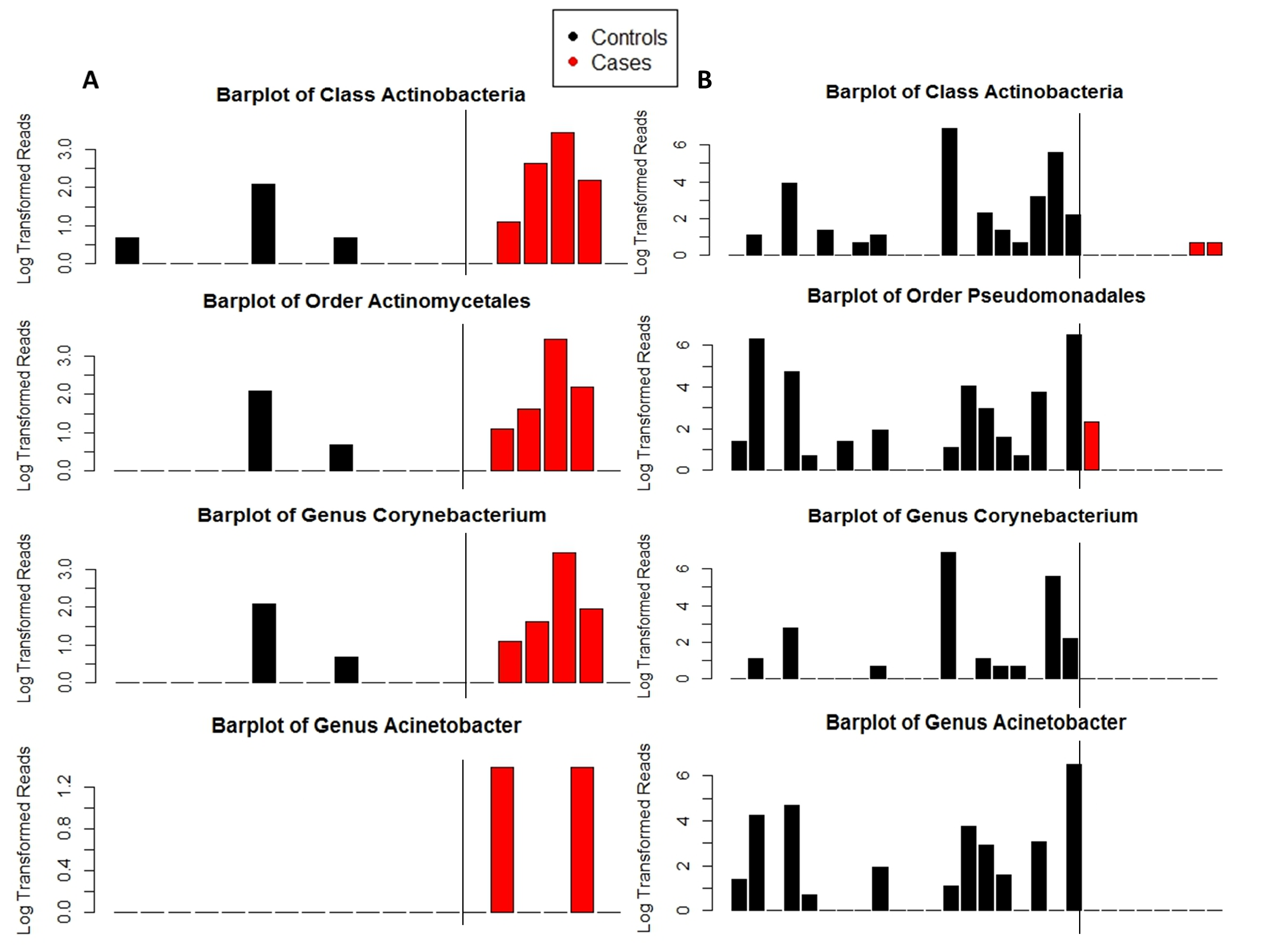

Supplement: S3 Fig — A) Taxa identified as different between Birmingham coagulase negative Staphylococcus cases and controls. Phyla Actinobacteria was also significantly different between cases and controls, it is not shown here because it has a correlation 0.9996 with class Actinobacteria and was visually indistinguishable. Family Corynebacteriaceae was also significantly different, it is not shown here because it has a correlation of 1 with genus Corynebacterium. Family Moraxellaceae was also significantly different between cases and controls, it is not shown here because it had a correlation of 1 with genus Acinetobacter. B) Taxa identified as different between Cincinnati coagulase negative Staphylococcus cases and controls. Phyla Actinobacteria was also significantly different between cases and controls, it is not shown here because it has a correlation 0.9996 with class Actinobacteria and was visually indistinguishable. Family Corynebacteriaceae was also significantly different, it is not shown here because it has a correlation of 1 with genus Corynebacterium. Family Moraxellaceae was also significantly different between cases and controls, it is not shown here because it had a correlation of 1 with genus Acinetobacter. (TIF) [file pone.0130604.s003.tif]
